# Adolescent sexual and reproductive health and rights policy for ethnic minority girls in Vietnam: a qualitative study with policy makers and service providers

**DOI:** 10.1080/16549716.2026.2619306

**Published:** 2026-02-13

**Authors:** Lia Burns, Hannah Pitt, Minh Duc Pham, Van Pham Thi Thanh, Peter Azzopardi, Samantha Thomas

**Affiliations:** a Institute of Health Transformation, School of Health and Social Development, Deakin University, Melbourne, Australia; b Global Adolescent Health Group, Burnet Institute, Melbourne, Australia; c Global Adolescent Health Group, Murdoch Children’s Research Institute, Melbourne, Australia; d School of Public Health and Preventative Medicine, Monash University, Melbourne, Australia; e ChildFund Vietnam, Programs, Hanoi, Vietnam; f The Kids Research Institute, Perth, Australia

**Keywords:** ethnic minority adolescent girls, sexual and reproductive health and rights, policy implementation, health inequity, Vietnam

## Abstract

**Background:**

Adolescent sexual and reproductive health and rights (ASRHR) policy has strengthened globally over the last three decades, but country-level barriers to implementation perpetuate health inequities for adolescent girls. In Vietnam, implementation of ASRHR policy remains challenged by persisting structural and socio-cultural issues and has yet to reduce the high prevalence of adolescent pregnancy in ethnic minority communities.

**Objective(s):**

To explore the perspectives of policy makers and service providers in Vietnam regarding the factors influencing the delivery of ASRHR policy for ethnic minority adolescent girls. The research examined professional opinions related to: (1) socio-cultural factors influencing the lives of adolescent girls in Vietnam; (2) current implementation of ASRHR policy for ethnic minority girls; and (3) strengthening ASRHR policy and service delivery for this population.

**Methods:**

Eleven key informant interviews were conducted across government and civil society, using semi-structured interviews via an online platform. Critical qualitative inquiry guided a reflexive approach to thematic analysis.

**Results:**

Three themes were constructed. First, conservative patriarchal values, parenting, and particular vulnerabilities of ethnic minority girls underpin health inequities. Second, universal responses to ASRHR policy weaken delivery of services and education in ethnic minority communities and are not responsive to emerging complexities. Third, strengthening ASRHR policy in Vietnam includes provincial level enhancements and better use of civil society resources.

**Conclusion:**

Key informants strongly supported ASRHR policy in Vietnam and called for improved policy level action to contextualize complexities and better use of available local resources. The recommendations could contribute to strengthening ASRHR policy in Vietnam.

## Paper Context

- **Main findings:** Important socio-cultural factors still affect the lives of adolescent girls in Vietnam and practical and feasible recommendations are offered to improve equitable sexual and reproductive health service delivery for ethnic minority adolescent girls.
- **Added knowledge:** This study addresses a neglected perspective on the determinants affecting positive sexual and reproductive health outcomes for adolescent girls in Vietnam, by exploring the experience and perspectives of key informants who implement adolescent sexual and reproductive health and rights policy in ethnic minority communities.
- **Global health impact for policy and action:** Improving the effectiveness of adolescent sexual and reproductive health policy in complex and diverse socio-cultural contexts would benefit from better use of local resources and closer policy-level attention to subnational factors.

## Background

### The global policy agenda for adolescent sexual and reproductive health and rights

Global policy action to address inequities in adolescent sexual and reproductive health and rights (ASRHR) for adolescent girls (10-19 years) remains a priority. Sustainable Development Goal 3.7 (SDG) [1] for reproductive health has specific indicators for adolescent girls [2] and the Global Strategy for Women’s, Children’s and Adolescent Health [3] promotes country policies that are in line with human rights principles [4]. Over the last three decades, increased global political will and financial investment have seen positive advancements in ASRHR outcomes for girls [5]. However, global figures showing gains in sexual and reproductive health and rights (SRHR) outcomes, mask persisting inequities related to specific determinants where women, children, or adolescents live [6]. Across the Asia-Pacific region, socio-cultural norms are a significant barrier to equitable ASRHR service delivery for adolescent girls [7]. Persisting health inequities for adolescent girls suggest that global and national-level policies may not prioritize efforts to address the drivers of these barriers [8, 9]. This scenario is now exacerbated, with past commitments weakening in countries where current ideology is diminishing female reproductive rights [10].

### National policy environment for adolescent sexual and reproductive health and rights in Vietnam

In Vietnam national legislation enables SRHR service coverage and protection [11] for adolescents and ethnic minority populations [12]. Globally, ASRHR policy agendas supported by strong legislation have decreased the frequency of unintended pregnancies [13]. This outcome has yet to be realized in Vietnam, where 7 million adolescent girls aged between 10 and 19 years old remain at risk of poor SRH outcomes, with national-level data showing inequities across geographic locations and ethnic groups [14]. The 2021 Multiple Indicator Cluster Survey (MICS) for Vietnam shows a total adolescent birth rate (ABR) of 42 births per 1000. Comparatively, an ABR of 115 per 1000 occurs in the Northern Midlands and mountainous regions where most ethnic minority groups live [15]. The 2021 MICS also provides comparable data for ethnic populations, showing an ABR of 28 per 1000 for the majority Kinh population and 92 per 1000 combined for the Tay, Thai, Muong, and Nung minority groups. Furthermore, the proportion of ethnic minority women aged 10–49 years old using contraception was only 51.1%, compared with an already low national average of 64.6% [16]. Within this context, macro level factors may be adversely affecting the national ASRHR agenda. For example, pro-natalist intentions by the GoV in response to concerns about the below replacement level fertility rate – 1.9 children per woman [17] – may be driving out-of-pocket costs for contraception [18] and excluding important adolescent and youth friendly health services (AYFHS) from health insurance programs [19], reducing access to SRH services for adolescent girls.

In 2020, the uncoordinated ASRHR policy platform in Vietnam [20,21] was brought together under the National Action Plan (NAP) on Reproductive and Sexual Health Care for Adolescents and Young People 2020–2025 [20]. The NAP outlines a comprehensive package of ASRHR services, including actions to remove critical barriers to positive SRH outcomes. This policy also acknowledges how structural risk factors contribute to poor SRH outcomes for adolescent girls, for example, when delivery of comprehensive sexuality education (CSE) in schools is unregulated, and the contraceptive needs of unmarried adolescents are not met [20]. Importantly, the NAP acknowledges social determinants of health inequities by recognising that restrictive patriarchal norms effect adult perceptions of adolescent sexuality [20]. Perceptions of sexuality often result in social stigma for unmarried adolescents seeking contraception, contributing to risky sexual practices and unintended pregnancy [22]. The NAP also notes that particular groups of adolescents are underserved, and recommends adapting policies for adolescents and young people 15 to 24 years old and ethnic minority groups [20]. Box 1, provides a summary of the two key NAP objectives addressing CSE and AYFHS with examples of policy actions from the official English translation of the policy [20].

**Box 1.** Summary of Objectives 2 and 3 from the NAP addressing SRH education and services.

The National Action Plan for reproductive and sexual health of adolescents and young people 2020–2025
**Objective 2 Comprehensive sexuality education** Awareness raising and behaviour change for the health care of adolescents and young people with parents, teachers and community, through communication, education and counselling activities, prioritising disadvantaged groups. **Example of policy actions** - Strengthen information, education and communication activities for the community on ASRHR. - Improve comprehensive health education and communication for health workers and the community, focusing on priority groups. **Objective 3 Adolescent and youth friendly health services** Increase access to and improve the quality of friendly health services for adolescents and young people. **Example of policy actions** - Review and update health care policies for adolescent and young people, using evidence and contextualising interventions. - Increase capacity of human health resources. - Strengthen multisectoral responses and mobilise more national and international funds.

The Ministry of Health is responsible for funding a multi-sectoral response to the NAP, with support and investment from partnerships with local civil society, socio-political organizations and international partners [20,21]. While the Government of Vietnam (GoV) aims for a pluralistic health system, Vietnam is characterized by ideological struggles between state and non-state actors, reducing the effectiveness of these resources [23]. In contrast, socio-political organizations, such as the Women’s Union [24] and the Youth Union [25], are aligned with the government, addressing local issues through local representation with broad national coverage [26]. Government data on the NAP outcomes are not currently available; therefore, improvements in policy implementation are unknown. Available data show that in 2019, 36% of adolescent girls aged 15 to 19 years old experienced unmet need for modern family planning methods [27]. In 2025, reporting continues on the high prevalence of adolescent pregnancy and induced abortion in ethnic minority communities [28,29].

### Adolescent sexual and reproductive health policy for ethnic minority populations in Vietnam

The unique needs of ethnic minority populations in Vietnam are addressed through the National Target Program (NTP) for Socio-Economic Development of Ethnic Minorities and Mountainous Areas for 2021–2030 period, Phase 1 2021 to 2025 [30,31]. Provincial-level governments are responsible for the implementation of the NTP supported by local civil society agencies, socio-political organizations and international partners. However, ASRHR education and services for ethnic minority adolescents remain the remit of the NAP. To date, the generic approach to implementing the NAP has yet to be formally adapted to address ASRHR for ethnic minority adolescents living in diverse socio-cultural contexts or experiencing emerging risks. For example, ethnic minority adolescent girls from a Tay community in northern Vietnam experience difficult interpersonal communication regarding SRH issues and increasingly rely on internet-based SRH sources, increasing risks related to misinformation and disinformation [32]. Consequently, the SRHR needs of this group are falling through a policy gap [5]. This scenario raises important questions about why country-level authorities are not committing to addressing known barriers to ASRHR policy implementation or are not willing to prioritise a response to the complexities of local demographics [33].

## Methods

### Objective

To explore the perspectives of policy makers and service providers in Vietnam regarding the socio-cultural and implementation factors influencing delivery of ASRHR policy for ethnic minority adolescent girls. This study was guided by three research questions:

RQ1. What do key informants perceive as the main socio-cultural factors affecting adolescent girls in Vietnam?

RQ2. How do key informants perceive current implementation of the ASRHR policy for ethnic minority adolescent girls in Vietnam?

RQ3. What recommendations do key informants offer to improve ASRHR policy and service delivery for ethnic minority adolescent girls in Vietnam?

### Study design

A critical qualitative inquiry approach [34] was used to explore the professional experience of key informants in policy making and service provision roles related to ASRHR policy in Vietnam. In their roles, key informants share a social justice commitment to make a difference in the lives of socially oppressed persons [34], and for which their experiences and views have limited representation in the evidence base for Vietnam. This study focuses on key informants’ subjective experiences of implementing ASRHR policy for adolescent girls in ethnic minority communities in Vietnam. The study applied a Big Q experiential approach to reflexive thematic analysis [35]. The study design and data analysis process was informed by a public health approach to the preventative needs of ethnic minority adolescent girls by exploring socio-cultural factors influencing delivery of ASRHR policy. This is consistent with the principles of The Ottawa Charter for Health Promotion in addressing determinants of health and health equity [36]. There were four female and two male members of the research team, who were public health researchers from Australia and Vietnam with expertise in ASRHR, community development, health promotion, and the determinants of health. Four members of the research team had previous or current work experience in Vietnam. An advisory group was established to guide this study, comprising members from civil society organizations in Vietnam and Australia.

### Study setting

The study was conducted from Australia via an online platform, with participants attending from Vietnam. All the data were analysed in Australia.

### Participants, sampling, and recruitment

This study sought key informant experts currently supporting ASRHR policy or in service delivery roles. Engaging with key informants provides in-depth opinions based on specific experience [37]. Purposive sampling techniques were used to identify key informants through the research teams’ professional networks across four stakeholder groups in Vietnam: the provincial health department in Thach An District, Cao Bang Province, local and international non-government organizations, and United Nations agencies. The first and fourth authors sent email invitations with plain language statements and consent forms to potential participants. Invitations were distributed in a staged approach to ensure a manageable number of participants were recruited at one time. The first author managed the email and phone call follow-up and registration schedules with support from the fourth author. Invitations were distributed to 17 individuals who were invited to participate as experts not as representatives of their organisation. A snowball sampling technique [38] was also used, asking participants to share the study information with colleagues they thought would be interested in participating. During recruitment, participants were given the option to either read, sign, and return the consent forms prior to the interview date, or discuss and sign prior to the interview commencing.

### Data collection

Eleven key informant interviews were conducted on MS Teams between November 2024 and March 2025 and lasted between 60 and 90 minutes. The semi-structured interview schedule was prepared in English and translated into Kinh (Vietnamese) language and key terminology was back translated to ensure intent was maintained between versions. The interview schedule was not pilot tested. Key informants were given the option to undertake the interview in English or Kinh with translation support. Participants were reminded of the voluntary nature of the interview, how their data may be used and their privacy protected. The first author only, conducted interviews in English. The first and fourth authors conducted interviews in Kinh, with the fourth author providing translation support. The fourth author led the questioning and translated each answer to the first author in English, who led prompting, which was then translated to the participant. The fourth author assisted in clarifying concepts and provided additional prompts. The interviews were video and audio recorded, and transcriptions were generated by MSTeams in English and Kinh. All transcriptions were checked against the original audio to ensure that the transcription and translation were clearly captured.

Broad interview topics addressed contextual information, socio-cultural determinants affecting adolescent girls, public health responses to ASRHR, and perspectives on improving the NAP response for adolescent ethnic minority girls. Interview questions also sought views on specific ASRHR services, such as AYFHS and CSE. Examples of these questions and prompts are in Box 2.

**Box 2.** Examples of questions and prompts from Key Informant interviews.

What do you think are the main socio-cultural factors influencing the SRHR of adolescent girls in Vietnam? Overall, do you think public health policy and service delivery could be more responsive to diverse socio-cultural traditions? (If yes, how? If no, why?) What about ethnic minority girls? Are there any issues particular to this population? Can you think of an example? Can you tell me about some of the programs (i.e. AYFHS and CSE) that are being offered to improve the SRHR of adolescent girls? What are their strengths and weaknesses? Where do you see opportunity for improvements? How?

### Data analysis

Reflexive thematic analysis provides a six-step process for an iterative approach to data analysis [35,39]. Qualitative data were entered and managed on Microsoft Excel spreadsheets accessible to the research team members for review. The first author started familiarization (Step 1) by cleaning the MSTeams generated transcription of the English and Kinh language translation, removing any identifying information, assigning unique identification code, and cross-checking with the audio file. The first author then re-read, took notes, and coded (Step 2) the data. The study research questions were used to guide and shape the analysis, but did not predetermine what was coded, with the researchers remaining open to different concepts across the dataset. Research team meetings considered initial coding, and the first author developed the themes (Step 3). During regular team meetings themes were reviewed (Step 4) and discussed before quotes and constructed themes were finalised (Step 5). During write-up, select quotes were slightly rephrased for better clarity in English. Three themes with three concepts were agreed upon by the team, which guided the final iterative write-up process (Step 6), with ongoing input from the team.

## Results

### General characteristics

There were a total of 11 participants from four stakeholder groups: nine from United Nations agencies, local and international non-government organizations, and two from the provincial department of health. The nine female and two male participants had an average of 16 years of professional experience (range of two to 25 years) working in ASRHR and with ethnic minority populations. Ten out of the 11 participants were from the Kinh ethnic group, with one from the Nung ethnic group.

Three themes were constructed from the data and aligned with the three research questions. Table 1 provides the themes, sub-themes and a selection of additional quotes related to themes.

**Table 1.** Themes, sub-themes and additional quotes.

Sub-themes	Quotes
**Theme One Complex socio-cultural contexts, values and expectations impact ASRHR outcomes for adolescent girls in Vietnam.**	
Cultural patriarchy is a strong influence in Vietnamese society Parents are an important factor for adolescent SRH and experience challenges in this role Adolescent girls face many complex contextual issues	*With ethnic minority people, they think that girls have no need to study higher because they are the labour force for the family or the caregiver of the children so they should just get married to provide labour force*. Service provider, Female, Participant 6 *The attitudes of the parents have a great influence on the behaviours of children and the adolescents*. Service Provider, Female, Participant 3. *Adolescents in boarding school do not get advice from family and parents. Everything they learn is through the peers and through the teacher. So, it can be a different dimension*. Service provider, Female, Participant 8 *It’s really different between urban and rural population, people in the urban areas are more modernised and have the opportunity to reach for more (SRH) information*. Service provider, Female, Participant 11
**Theme Two ASRHR implementation strategies for adolescent ethnic minority girls in Vietnam are not always aligned with the realities of young people’s lives**.	
ASRHR policy and programs are not addressing the realities of implementation The current approach to AYFHS is not locally relevant The current approach to CSE is under resourced and not locally relevant Online SRH resources are an opportunity and a threat	*I think we still can improve the policy, the government wants to make the change, but it’s not easy. I think they are working on that and for me I also don’t know how to improve because we need to address the need from the culture and the differences*. Policy maker, Male, Participant 2 *In these areas, the health facility can provide services, but they don’t really care whether girls at the age 13 or 14 come for those services, and for the girls in those area, they don’t care very much about like pregnancy*. Service provider, Female, Participant 6 *We also work with some schools, and we see that even teachers in school, they also expressed that the sexuality education is not priority*. Service provider, Female, Participant 4 *With social media information, sometime reliable, sometime not reliable, but I think that with their smart phone now, people can find information from that and also they can learn by themselves and that is a really good thing*. Service provider, Female, Participant 10 *The government or the NGO, they do not meet the demand for the way the adolescent want information. They choose the traditional way, but the adolescent may not be interested*. Service provider, Female, Participant 4
**Theme Three Investing in local solutions to support and sustain the implementation of specific ASRHR interventions**.	
Government investment for local adaptation of SRH programs Potential for more civil society engagement at the community level Civil society and mass organisations can be a stable resource for SRH program delivery	*I think it would require a national framework to support adolescent health because when there’s a framework there can be budget allocation for that, there could be partners identified in key roles and supporting role and there will be monitoring and evaluation. There will be national program to see how things are being implemented and there will be the review by the National Assembly to see how it works, where it works well or not well. What is the gap and how to do it*. Service provider, Female, Participant 9 *We have been piloting many, many programme for many years. Actually, after piloting, it should be adapted and applied in other places*. Service provider, Female, Participant 6 *I think the resource is underused and is not used effectively. For example, the Woman Union, which is focusing on the marriage woman. They also just focusing on some daily life issues, like CPI, and cost of living*. Service provider, Female, Participant 4 *Of course, regarding reproductive health for girls with disability and girl in general we should have a multi stakeholder to support them. The government and the local CSO. Even with the big providers and mass organization, they still can support them, and I think the government is to play coordinator better than the other*. Service provider, Female, Participant 8

### Theme One: complex socio-cultural contexts, values and expectations impact ASRHR outcomes for adolescent girls in Vietnam

Participants shared that conservative cultural values and the effects of diverse vulnerabilities were significant contextual issues affecting adolescent girls in Vietnam. All participants shared the view that conservative Asian culture and Confucianism impacted ASRHR outcomes. One participant explained that there was a ‘patriarchal culture here’ where women were expected to produce children and care for the family and young people had a ‘low voice’ in societal and familial spaces. Another participant shared that conservative culture supported a dominant role for males who made decisions about female health, perpetuating gender inequities. Most participants perceived that the prevailing culture influences the attitudes of adults towards adolescent sexuality and inhibits interpersonal communication.

Second issue is the customs and tradition, people think talking about and discussing about this [SRH] topic is evil and not good, they think it’s shameful to think about this. They don’t see it is a physical need of people. Service provider, Female, Participant 11

Participants shared that parenting was an important determinant for adolescents learning about ASRHR, and that the attitude of parents was very influential. All participants agreed on the importance of ‘young people learn(ing) from the older generation.’ However, participants also shared views on the range of challenges experienced by parents across rural and urban locations. Participants perceived that parents were generally influenced by cultural patriarchy, with high ideals for educational attainment often the basis of family conflict. Based on their experience, participants explained how work and employment commitments for parents inhibited time spent with their adolescent children and that family cohesion within the urban family was more ‘loose’ than the rural families. However, another participant felt that urban families could teach their children better than rural families due to perceived higher education levels but felt that parents were important to adolescent sexual development and that more support should be provided for parents.

No, parents are not involved when we go to school for SRH activities with grade 7,8 and 9, I think it would be better to inform and have parents to join the SRH activities. Service provider, Female, Participant 10

Based on their experiences, all participants shared insights into the range of vulnerabilities experienced by adolescent girls living in ethnic minority communities. One participant explained that services implemented under the NAP did not address the needs of adolescent girls with disabilities, especially those living in ethnic minority communities. A few participants were also concerned about ethnic minority adolescent girls from isolated rural villages attending boarding schools to access high school education. Participants explained that boarding schools provided poor accommodation with limited adult supervision and no SRH services, which could result in many ‘unwanted pregnancies.’ Another participant felt that it was the ethnic minority girls from the most geographically isolated areas that were most vulnerable because in these communities, early sexual activity, pregnancy, or marriage was accepted as natural. One participant shared that in very isolated communities highly dependent on agriculture, it was common for families to be considered a labour force, so marriage and children were a priority. This participant also shared that for adolescent girls in these areas, if they were not married before they reach 18 years they were valued less and considered an ‘old maid.’

Ethic minority people who live in mountainous areas, most work on the farm so if they have more people to work then it is good for the family. That is why all the village and family encourage their son to marry early. For the girls, if they are 14 or 16 years old without any people interested in getting married to them, they will become an old maid at the age of 18 that is why, they have less value than the other girl, that is why they have to get married early. Service provider, Female, Participant 6

### Theme Two: ASRHR implementation strategies for adolescent ethnic minority girls in Vietnam are not always aligned with the realities of young people’s lives

Participants shared a range of perspectives on the implementation of the current NAP, with particular concerns for ethnic minority girls. Overall, in their experience, the NAP was comprehensive but only relevant for the general population, with policy guidance and programming unresponsive to the nuances of ‘disability, age, gender or ethnicity.’ Other participants felt that adolescents were underserved, explaining that ASRHR services are ‘just not available’ and that SRH promotion for adolescents was rare. Participants felt positive that the NAP had been developed but also felt that implementation was a big challenge for the government, and reliance on local and international NGOs was significant. When asked about the policy response for ethnic minority populations, one participant thought that grouping all ethnic minority populations together made the policy weaker and did not address those who were most at risk of poor SRH outcomes. One participant felt that culture and diversity of needs were persistent barriers to delivering the NAP. They further noted that adapting policies at the provincial level remained a challenge related to the capacity and resources available at the provincial-level Department of Health.

I think the big problem is the capacity. So, we need the implementation level to be more customized, but it depends on capacity of the implementer and the readiness of the resources and facilities they have in the Provinces. Policy maker, Male, Participant 2

The current approach to delivering the NAP was not responsive to prevailing attitudes towards ASRHR or health-seeking behaviour in ethnic minority communities. Participants felt that many ethnic minority communities experienced low health literacy, resulting in lower utilization of the AYFHS and health services overall. One participant explained that, in some ethnic minority areas, the demand for SRH services was very low, as the community did not consider early pregnancy to be a problem. They also shared that geographical isolation, out-of-pocket costs for contraception, and low trust in commune- and district-level health services continued to prevent the uptake of SRH services in these communities. The issue of the declining national budget for ASRHR services was of considerable concern to all participants. Their view was that limited resourcing resulted in insufficient ongoing training for health workers and reduced the occurrence of community-based service delivery and health promotion for adolescents.

But recently, the government, they reduced significantly the budget for implementation of health promotion. Because, yeah, so the community health officers, they don’t have many resources to carry out the health promotion. Policy maker, Female, Participant 3

Participants also felt that the current approach to implementing the CSE curriculum was insufficient. Participants shared that, in their professional opinion, school-based delivery of the CSE curriculum was not a priority for the education system and felt that efforts to integrate select content into existing subjects were unsuccessful. Participants shared their experiences with teachers during fieldwork, explaining that they were undertrained and overburdened with current demands and that sexual education was ‘not a priority.’ One participant also thought the didactic style of the current education system was a problem for CSE delivery and education overall was not ‘in a model, or best practice way.’ Other participants shared that although the government advocates that the CSE was designed for everyone, it was not suitable for girls with disabilities or for adolescent girls out of school. Two participants shared that they had never heard of CSE before and did not know what the education system did to address the SRH of adolescent girls.

I receive annual training on SRH; however, I haven’t heard about comprehensive curriculum on this. Service provider, Female, Participant 11

Internet-based online SRH sources were perceived by participants as an opportunity and a threat to adolescent populations. Participants felt that the government needed a formal response to unofficial online SRH information, and greater effort to meet this populations demands for safe and reliable online sources. Other participants were concerned that there was an over reliance on the internet as a proxy for SRH information and education, in lieu of unsatisfactory public services. One participant explained that in their work, they had observed many adolescent girls who experienced online sexual abuse and misuse of their images. Other participants shared that online SRH information had much potential and was the best way for girls living with disabilities to receive information. However, one participant was not convinced that online support for SRH was possible for some ethnic communities, where access to data was scarce, adolescents were unable to read the Kinh language, and where male adolescents were given priority for access to smartphones.

Because having the smartphone, even if they did, they don’t have a monthly subscription for the internet and access to data, so they have to go somewhere to access the internet, if they want access they have to go to common places like the health facility or commune for free Wi-Fi. And boys often have priority for smartphone rather than the girls, if they have the phone they use TikTok to make friends not accessing information. Reading documents in Kinh language, they can speak but not read the Kinh language. Other methods (are better) for circulating information, providing vender information or some other ways that our program have been working on, like providing a clinic for them to see, not using the internet for SHR information. Service provider, Female, Participant 6

### Theme Three: investing in local solutions to support and sustain the implementation of specific ASRHR interventions

All participants expressed urgent need for specific policy actions and financial investments for ASRHR. One participant detailed how a national framework with formal budget allocation, formalised partnerships and coordination, with monitoring by the National Assembly were necessary. Participants shared their experience of limited ministry and donor coordination, which contributed to protracted ‘piloting’ of projects that, even when successful, were rarely scaled up. Other participants called for more funding for health worker training and ‘multiple’ public ASRHR campaigns necessary to change people’s attitudes about adolescent sexuality. One participant called for task shifting from doctors to midwives to enable important, long-lasting contraceptive services to be provided at the commune level.

I would like to extend the scope of works for the service provider like the midwife in the community because you know at the commune level, in many, many commune health stations they don’t have doctor. They have only midwife, but they are very, very skilful midwife but they are not allowed to provide long term methods. And yeah, I would like to extend the scope of work. or the service provider, especially in the at the Commune Health station. Service provider, Female, Participant 5

All participants felt that local and international NGO’s have the potential for greater impact at the community level. Participants shared that these organizations were an important resource, bringing innovation and mobilization for community development. Participants felt that NGOs had a role in supporting provincial authorities to adapt ASRHR policies for local needs and mobilize resources for the delivery of CSE in schools. Some participants shared that international NGOs were highly valued by the government and needed to be more strategic in demonstrating how they are responding to community needs. This participant also felt that local NGOs would benefit from better partnerships and capacity building with other development partners.

They (local NGO’s) are not supported by other development partners, for example, we try to select best practice from them and advocate for them, but not all of them have a good partnership with such a way, because we’re talking about new relation. But overall, I say that introduction of NGO in Vietnam is a very important way to address sexuality issues. And it has happened; we do support NGOs. Policy maker, Male, Participant 2

Most participants shared that socio-political organizations were an important but currently underutilized resource at the community level. Participants felt that since funding from the international community was reduced, the Women’s Union in particular was only active in advocacy at the national level. However, in their experience, the Women’s Union only ever had limited activity in ethnic minority areas. One participant noted that perhaps they were not as influential as they used to be because ‘society has changed like water.’ Participants also recognized that there was an opportunity for organizations like the Youth Union to play a larger role in effective ASRHR initiatives because they have a mandate to work with adolescents. However, there was recognition that their membership was low and that they currently only did events during school holidays. Participants shared the view that socio-political shifts and funding changes requires a different approach from socio-political organizations. One participant suggested that the government should consider merging these organizations to strengthen their role in community development, and another imagined the great potential of these organizations if they were funded.

You see that they are not really active, but they still have their own systems from the central to the village. So they are, of course, very important. We have youth union, we have women’s union, there are also family associations as well. So, do you see how these organisations have great potential to be supporting the health of the community if they were funded? Policy maker, Female, Participant 3.

## Discussion

This study aimed to explore the professional experiences of key informants implementing ASRHR policy, regarding the socio-cultural context that affects adolescent girls, their views and perspectives on policy implementation for ethnic minority girls, and how the policy response could be improved. There are three main areas for discussion.

First, there were fundamental socio-cultural issues affecting adolescent girls and additional complexities for those in ethnic minority communities. Prevailing cultural patriarchy based on Confucianism remains highly influential in the social hierarchy across all social strata in Vietnam, with females and adolescents subordinate to males and restrictive attitudes toward female sexuality [40]. This cultural context perpetuates gender inequality, influences adult attitudes and communication regarding adolescent sexuality, and is also perceived to be the basis of challenging and complicated parental experiences. The Global Gender Gap Report 2021 states that the Confucian sphere of influence is significant for approximately 1.5 billion people, or 20% of the world’s population, across countries such as China, South Korea, and Vietnam, where gendered inequities for women and girls’ health and wellbeing are widespread [41]. However, in secular Vietnam, Confucian philosophy is blended with other values such as Buddhism, Christianity, Taoism, and ancestor worship [42], indicating the range of influences at play in determining social values. Research also shows how these values are transmitted intergenerationally through family rules and teachings, contributing to the formation of local gender norms and power dynamics, which play an important role in adolescent sexual development [41,43].

A range of practical issues in accessing basic education is increasing risks for the SRH of ethnic minority adolescent girls. Semi-boarding ethnic schools provide weekday accommodation and care for geographically isolated students who may return home on weekends. This education policy contributes to universal access to education but jeopardizes the health and well-being of adolescent girls living away from family care. To date, robust evaluation or research on health outcomes in ethnic minority boarding schools is unavailable; however, a study by UNICEF [44] reported that ethnic minority students are at an increased risk of sexual abuse due to inadequate adult supervision and poor accommodation infrastructure. This report is consistent with a 2020 mixed-methods study on the operations of semi-boarding ethnic schools, which found that teaching CSE was difficult and that the student population needed this type of education [45].

Implications for the SRH of adolescent girls from cultural patriarchy and educational attainment in Vietnam, are consistent with factors addressed in the literature across the Asia-Pacific region. The research highlights that reproductive health and gender equity policies fail to meet the needs of women and girls based on contextual factors where they live and are educated [46]. In Vietnam, the NAP and NTP policy agenda recognizes the specific needs of these population groups [20], but community-level implementation, coordination, and funding levels are not sufficient for effective policy outcomes. National-level advocacy must call for adequate funding to support community level initiatives that disrupt the drivers of gender inequality, increase parental understanding of ASRHR, and increase efforts to include SRH education and services in ethnic minority boarding schools.

Second, key informants shared two main aspects of concern regarding implementation of the ASRHR policy for ethnic minority girls in Vietnam. The generalist approach to ASRHR policy is not responsive to demographic determinants of age, gender, disability, and ethnicity and emerging issues related to the digital determinants of health remain unaddressed. Contextualizing policy at the subnational level is a significant part of the solution but is hampered by insufficient financial and human resources, limited understanding of the actual needs of ethnic minority adolescents, and little political will and capacity. In many ethnic minority communities, ASRHR services need to respond to limited health and digital literacy, low trust in public health services, normative views on early pregnancy, and those attending boarding schools. These service delivery needs are consistent with global calls to expand the adolescent health agenda in a progressive and strategic manner [47]. This is particularly relevant when online ASRHR resources are emerging as proxy information, education, and service delivery options. In this case, new risks related to misinformation, disinformation, and online abuse are increasing for ethnic minority adolescent girls.

The scenario of persistent and emerging risks to ASRHR has been documented in other Southeast Asian countries with significant ethnic minority populations. In the Philippines, the ASRHR policy response is also non-responsive to the needs of certain groups with limited and poorly coordinated public resources [48], resulting in a gap between policy and implementation [49]. The SDG Leave No One Behind agenda states that the SDGs will not be achieved, while significant and vulnerable population groups do not benefit from policies, programs, or investments aimed at improving health outcomes. This call is also noted in the second Lancet Commission, A Call to Action [50] which considers ASRHR policies as the foundational environment for continuous improvement. For countries like Vietnam, with significant urban-rural divide in terms of poverty and access to public services, reviewing and updating the NAP to improve service delivery to marginalized populations is urgent. Clarity on a way forward to comprehensive ASRHR for all adolescents may require a paradigm shift, one that identifies persistent and emerging equity challenges and brings social change with more transformational initiatives [51]. In this light, there is an urgency for the NAP to be updated, with improved leadership for subnational responses, and greater participation from adolescent girls to engage in ASRHR policy and address emerging new challenges, especially in ethnic minority communities.

Third, at the end of the NAP 2020–2025 implementation period, it is timely to evaluate and assess the national policy approach. The NAP could be enhanced by an overarching national framework with budgetary allocation, formal monitoring and evaluation, clearer accountability, and stronger national and subnational coordination. The national framework model was successful for maternal and child health policy, where funding and coordination since 2011 have increased antenatal care visits, under five immunizations, and reduced infant mortality [52]. Linkages with NTP programs could mitigate the challenges of the current general approach and bring equitable service delivery to ethnic minority populations. Inspired by Marmot’s principle of proportionate universalism [53], policy coordination could deliver targeted services at a scale and intensity proportionate to the degree of need with a national framework supporting a comprehensive assessment of need for ethnic minority adolescents [54].

**Figure 1.**
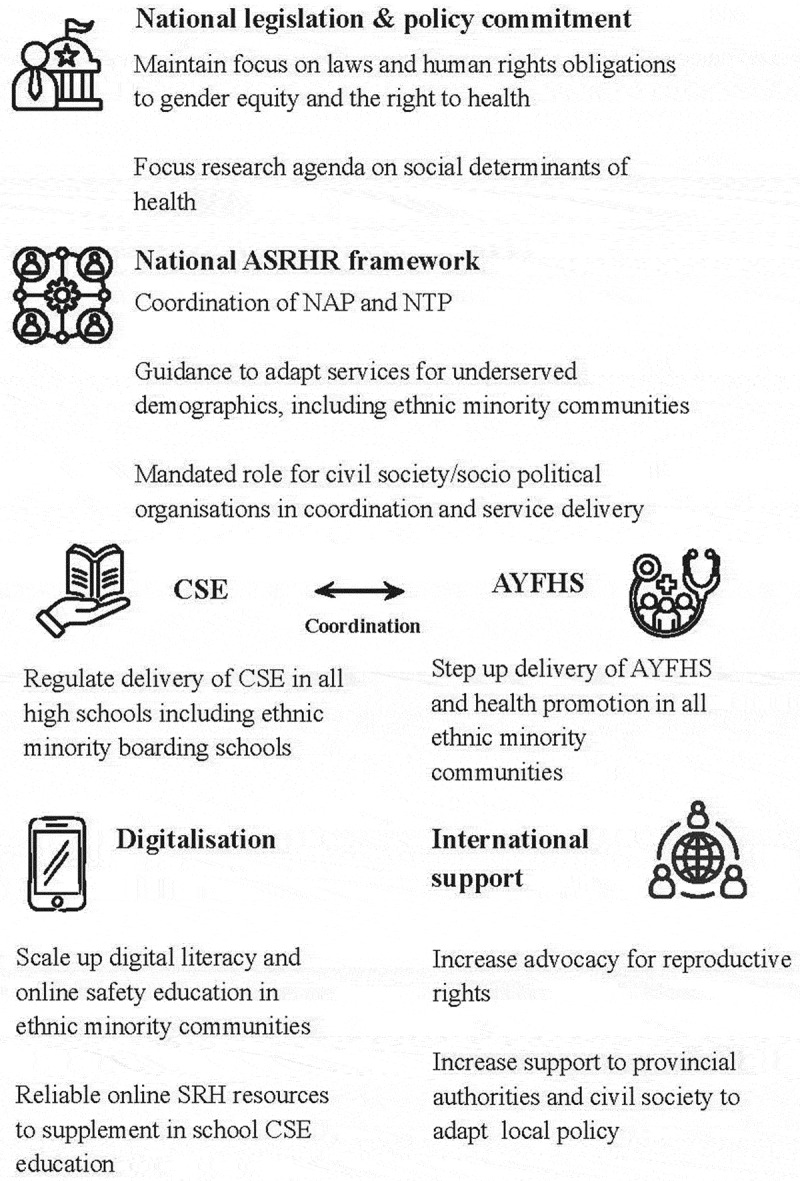
Recommendation for ASRHR policy development.

To achieve this, policy coordination and leadership would benefit from strengthening the relationship and engagement with socio-political and non-government organisations. Together, these stakeholders can support monitoring and data collection to inform the review and revision of the national-level ASRHR policy, and support adaptation and implementation at the subnational level. This would also be a timely, cost-effective, and meaningful contribution to decolonizing public health and localizing the development agenda. An opportunity exists for international organizations to reimagine their strategic value, where new donors and funding models may not focus on rights-based needs. Global calls seek prioritization of health inequities that undermine social foundations [55] and international agencies could prioritize advocacy for national funding and reproductive rights, support in-depth formative understanding of susceptible groups, and champion the resourcing of community initiatives to monitor health inequities. These notions are consistent with significant literature from UN agencies on structural barriers affecting ASRHR policy [33,56–58] which promote practical and successful engagement between civil society and communities in policy implementation, monitoring, and evaluation [59,60]. The GoV has already shown positive intent by delivering a cohesive NAP 2020 to 2025. Committing the same to the evaluation, review, and revision of the NAP and NTP to respond more closely to subnational issues using local resources for contextual relevance are feasible options with a large return on investment. Based on key informants’ professional views, Figure 1 presents’ recommendations for ASRHR policy development for the GoV and relevant stakeholders.

## Limitations

This study interviewed 11 key informants, the majority of which were from NGO’s with only two GoV staff members from the provincial level. Therefore, the views and opinions shared were mostly from the perspective of civil society actors, and government perspectives from higher levels of government were not gathered. The participation of more GoV staff at the national level may have shared different perspectives on policy implementation that were not considered in this study. Ten participants were from the Kinh ethnic group, and one was from the Nung ethnic minority group, providing insight into the small number of ethnic minority professionals in policy or practitioner roles working with ethnic minority populations. Broader experiences from higher levels of government or additional health professionals from ethnic minority groups may have been found with additional participants, had time and resources been available.

## Conclusion

This study found that key informants delivering the NAP 2020–2025 were supportive of policy objectives and shared consistent views on how to improve implementation in ethnic minority communities. Key informants acknowledged how entrenched cultural patriarchy continues to reduce the effectiveness of policy implementation nationally, and that challenges are exacerbated in ethnic minority communities. Local and international NGOs are underutilized, and in collaboration with the GoV, must reconsider their mandate in a changing social and development context. Policy review and development is urgent and should prioritize stable funding, cohesive policy guidance between the NAP and NTP, inter-ministerial coordination, and attention to emerging determinants of health.

## Supplementary Material

Reporting_checklists_BraunClarke_COREQ.docx

## Data Availability

Data that support the findings of this study may be available upon request from the senior author. The data are not publicly available because of privacy commitments to the participants and the guidelines of the GoV Personal Data Collection.
