# Supplementary material for: Adolescent sexual and reproductive health and rights policy for ethnic minority girls in Vietnam: a qualitative study with policy makers and service providers
Source: Glob Health Action. 2026 Feb 13;19(1):2619306. doi: 10.1080/16549716.2026.2619306 (PMC12912207; doi:10.1080/16549716.2026.2619306)
Supplement: Reporting_checklists_BraunClarke_COREQ.docx [file ZGHA_A_2619306_SM8524.docx]

**Braun and Clarke & COREQ Checklists**

**Braun & Clarke 15-point Thematic Analysis Checklist**

| Process | No. | Criteria | Response |
| --- | --- | --- | --- |
| Transcription | 1 | The data have been transcribed to an appropriate level of detail, and the transcripts have been checked against the tapes for ‘accuracy’ | All 11 transcribed audio files were translated and then checked with original audio for accuracy of transcription and translation. |
| Coding | 2 | Each data item has been given equal  attention in the coding process | Yes, coding was conducted following close analysis and consideration of all quotations. |
|  | 3 | Themes have not been generated from a few vivid examples (an anecdotal approach), but instead the coding process has been thorough, inclusive and comprehensive | All e11 translated files were reviewed and coded individually and then collated as a full dataset and coding reviewed to construct the themes. This process was thorough, inclusive of all data and comprehensive due to the reflexive review and feedback from the research team. Each theme comprised of three to four main codes created from across all quotations. |
|  | 4 | All relevant extracts for all each theme  have been collated | Yes, in Microsoft Excel. |
|  | 5 | Themes have been checked against each  other and back to the original data set | Yes, as part of the reflexive process |
|  | 6 | Themes are internally coherent,  consistent, and distinctive | Yes, checked as part of the reflexive process. |
| Analysis | 7 | Data have been analysed- interpreted, made sense of- rather than just  paraphrased or described | Yes, the themes and discussion reported show the analysis. |
|  | 8 | Analysis and data match each other- the  extracts illustrate the analytic claims | The data and analysis are consistent and compatible with the chosen quotation extracts used to support the analytic claim. |
|  | 9 | Analysis tells a convincing and well-  organised story about the data and topic | Yes, the analysis presented is across three thematic areas building a logical and coherent story. |
|  | 10 | A good balance between analytical narrative and illustrative extracts is  provided | Yes, quotations from the dataset are used as illustrative extracts set within the results analysis. |
| Overall | 11 | Enough time has been allocated to complete all phases of the analysis adequately, without rushing a phase or  giving it a once-over-lightly | Yes, the data analysis process was in-depth and thorough and reviewed with the full research team regularly. |
| Written report | 12 | The assumptions about, and specific approach to, thematic analysis are clearly  explicated | Yes, this detail is explained in the methods section. |
|  | 13 | There is good fit between what you claim you do, and what you show you have done- i.e. described method and reported  analysis is consistent | Yes, the described methods are consistent with study aim and the results reported. |
|  | 14 | The language and concepts used in the report are consistent with the  epistemological position of the analysis | Yes. |
|  | 15 | The researcher is positioned as *active* in the research process; themes do not just  ‘emerge’ | Yes, the researcher has taken an active role in the construction of the themes. |

Reference: Adapted from Braun V, Clarke V. Successful qualitative research: a practical guide for beginners. London: SAGE Publications Ltd; 2013.

# COREQ (Consolidated criteria for Reporting Qualitative research) Checklist

A checklist of items that should be included in reports of qualitative research. You must report the page number in your manuscript where you consider each of the items listed in this checklist. If you have not included this information, either revise your manuscript accordingly before submitting or note N/A.

| **Topic** | **Item No.** | **Guide Questions/Description** | **Reported on**  **Page No.** |
| --- | --- | --- | --- |
| **Domain 1: Research team**  **and reﬂexivity** | | | |
| *Personal characteristics* | | | |
| Interviewer/facilitator | 1 | Which author/s conducted the interview or focus group? | page 7 |
| Credentials | 2 | What were the researcher’s credentials? E.g. PhD, MD | page 6 |
| Occupation | 3 | What was their occupation at the time of the study? | page 6 |
| Gender | 4 | Was the researcher male or female? | page 6 |
| Experience and training | 5 | What experience or training did the researcher have? | page 6 |
| *Relationship with*  *participants* | | | |
| Relationship established | 6 | Was a relationship established prior to study commencement? | page 6 |
| Participant knowledge of  the interviewer | 7 | What did the participants know about the researcher? e.g. personal  goals, reasons for doing the research |  |
|  |  |  | page 6 |
|  |  |  |  |
| Interviewer characteristics | 8 | What characteristics were reported about the inter viewer/facilitator?  e.g. Bias, assumptions, reasons and interests in the research topic |  |
|  |  |  | page 6 |
|  |  |  |  |
| **Domain 2: Study design** | | | |
| *Theoretical framework* | | | |
| Methodological orientation and Theory | 9 | What methodological orientation was stated to underpin the study? e.g. grounded theory, discourse analysis, ethnography, phenomenology,  content analysis |  |
|  |  |  | page 6 |
|  |  |  |  |
| *Participant selection* | | | |
| Sampling | 10 | How were participants selected? e.g. purposive, convenience,  consecutive, snowball |  |
|  |  |  | page 6, 7 |
|  |  |  |  |
| Method of approach | 11 | How were participants approached? e.g. face-to-face, telephone, mail,  email |  |
|  |  |  | page 6 |
|  |  |  |  |
| Sample size | 12 | How many participants were in the study? | page 8 |
| Non-participation | 13 | How many people refused to participate or dropped out? Reasons? | page 9 |
| *Setting* | | | |
| Setting of data collection | 14 | Where was the data collected? e.g. home, clinic, workplace | page 7 |
| Presence of non-  participants | 15 | Was anyone else present besides the participants and researchers? |  |
|  |  |  | n/a |
|  |  |  |  |
| Description of sample | 16 | What are the important characteristics of the sample? e.g. demographic  data, date |  |
|  |  |  | page 7 |
|  |  |  |  |
| *Data collection* | | | |
| Interview guide | 17 | Were questions, prompts, guides provided by the authors? Was it pilot  tested? | page 7 |
|  |  |  |  |
| Repeat interviews | 18 | Were repeat inter views carried out? If yes, how many? | n/a |
| Audio/visual recording | 19 | Did the research use audio or visual recording to collect the data? | page 7 |
| Field notes | 20 | Were ﬁeld notes made during and/or after the inter view or focus group? | n/a |
| Duration | 21 | What was the duration of the inter views or focus group? | page 7 |
| Data saturation | 22 | Was data saturation discussed? | n/a |
| *Transcripts returned* | *23* | *Were transcripts returned to participants for comment and/or* | *page 7* |
|  |  |  |  |

|  |  | correction? |  |
| --- | --- | --- | --- |
| **Domain 3: analysis and**  **ﬁndings** | | | |
| *Data analysis* | | | |
| Number of data coders | 24 | How many data coders coded the data? | page 8 |
| Description of the coding  tree | 25 | Did authors provide a description of the coding tree? |  |
|  |  |  | n/a |
|  |  |  |  |
| Derivation of themes | 26 | Were themes identiﬁed in advance or derived from the data? | page 8 |
| Software | 27 | What software, if applicable, was used to manage the data? | page 8 |
| Participant checking | 28 | Did participants provide feedback on the ﬁndings? | page 8 |
| *Reporting* | | | |
| Quotations presented | 29 | Were participant quotations presented to illustrate the themes/ﬁndings?  Was each quotation identiﬁed? e.g. participant number |  |
|  |  |  | pages 9 to 15. |
|  |  |  |  |
| Data and ﬁndings consistent | 30 | Was there consistency between the data presented and the ﬁndings? | pages 10 to 18 |
| Clarity of major themes | 31 | Were major themes clearly presented in the ﬁndings? | pages 10 to 15 |
| Clarity of minor themes | 32 | Is there a description of diverse cases or discussion of minor themes? | n/a |

Developed from: Tong A, Sainsbury P, Craig J. Consolidated criteria for reporting qualitative research (COREQ): a 32-item checklist for interviews and focus groups. *International Journal for Quality in Health Care*. 2007. Volume 19, Number 6: pp. 349 – 357

**Once you have completed this checklist, please save a copy and upload it as part of your submission. DO NOT include this checklist as part of the main manuscript document. It must be uploaded as a separate file.**
